# Recommendations for a better patient care in daily clinical practice: a joint perspective of ESCAP and ECAP

**DOI:** 10.1007/s00787-012-0323-4

**Published:** 2012-09-22

**Authors:** Aribert Rothenberger, Ruud Minderaa

**Affiliations:** 1 Child and Adolescent Psychiatry, University Medicine Göttingen, Von-Siebold-Str. 5, 37075 Göttingen, Germany; 2 Child and Adolescent Psychiatry, University Medical Center Groningen, Hanzeplein 1, 9713 GZ Groningen, The Netherlands

The ESCAP (European Society for Child and Adolescent Psychiatry) and the ECAP-Journal have at least three joint ventures.

First, strengthening research in Child and Adolescent Psychiatry (CAP), second, developing and publishing evidence-based clinical guidelines for better patient care and third, discussing public health issues related to CAP.

Today, our joint perspective is related mainly to clinical care and public health from three different European countries.

Two statements signalize that the discussion about the best way for patient care has to be guided by empirically and critically evaluated evidence-based research. Both author groups show clearly, that on the way to improvement of patient care old fashioned principles need to be overcome. Especially, the manifest from France [1] highlights, that there is a growing group of open-minded clinicians and researchers pointing to the difficult situation of French CAP, while installing some necessary changes. Hopefully, a positive echo will spread out to many others in the country.

The Consensus Statement of our English colleagues [2] is recommending several practical issues to further develop ADHD services within the National Health Services across the UK. It remains to be determined if these recommendations and the spectrum of participating specialists might be a model also for other European countries.

Finally, the Spanish authors from Madrid [3] delivered some data on longitudinal trends concerning the changes in frequency of CAP disorders. Although this reflects the special situation in Spain/Madrid only, some similarities and differences compared to other longitudinal studies on time trends and mind trends [4] may be of value for reflection to own practical experience.

In sum, at many places in Europe more and more qualified basic provision research in CAP is ongoing showing that child psychiatrists are heavily engaged in the quality of care and wellbeing of their patients.
